# Deciphering role of FGFR signalling pathway in pancreatic cancer

**DOI:** 10.1111/cpr.12605

**Published:** 2019-04-03

**Authors:** Xiaodiao Kang, Zeng Lin, Minhui Xu, Jun Pan, Zhi‐wei Wang

**Affiliations:** ^1^ Department of Orthopaedics Surgery The Second Affiliated Hospital and Yuying Children's Hospital of Wenzhou Medical University Wenzhou China; ^2^ Center of Scientific Research The Second Affiliated Hospital of Wenzhou Medical University Wenzhou China; ^3^ Department of Pathology, Beth Israel Deaconess Medical Center Harvard Medical School Boston Massachusetts

**Keywords:** FGF, FGFR, pancreatic cancer, target, therapy

## Abstract

Recently, fibroblast growth factors are identified to play a vital role in the development and progression of human pancreatic cancer. FGF pathway is critical involved in numerous cellular processes through regulation of its downstream targets, including proliferation, apoptosis, migration, invasion, angiogenesis and metastasis. In this review article, we describe recent advances of FGFR signalling pathway in pancreatic carcinogenesis and progression. Moreover, we highlight the available chemical inhibitors of FGFR pathway for potential treatment of pancreatic cancer. Furthermore, we discuss whether targeting FGFR pathway is a novel therapeutic strategy for pancreatic cancer clinical management.

## 1 INTRODUCTION

Pancreatic cancer is one of the common malignancies in human worldwide. In fact, 56 770 new cases of pancreatic cancer and 45 750 deaths have been expected this year in the United States.^1^ More than 400 000 deaths annually due to pancreatic cancer are observed in the worldwide.^2^ Pancreatic cancer is the third leading cause of cancer death behind lung cancer and colon cancer in the United States in 2018. However, deaths from pancreatic cancer are predicted to be the second leading cause of mortality in the United States by 2030.^3^ The causes of pancreatic cancer are still unclear, although accumulating evidence has suggested that pancreatic cancer occurrence is associated with several factors such as smoking, drinking, coffee consumption, high fat and high protein diet, and genetic background. In addition, the patients with diabetes and chronic pancreatitis have high risk for developing pancreatic cancer.^4^, ^5^ In contrast to the increase in survival for most cancer types, the 5‐year relative survival rate for pancreatic cancer is about 8% in the United States. One of the reasons is that pancreatic cancer is often diagnosed at a distant stage, which has 3% for the 5‐year survival rate.^6^ Because the early symptoms of pancreatic cancer are same as gastric disease such as upper abdominal discomfort and loss of appetite, most of the patients with pancreatic cancer often exhibit locally invasion or metastatic tumour when they are diagnosed.^7^ About 95% of pancreatic cancer cases are adenocarcinoma, known as PDAC (pancreatic ductal adenocarcinoma), which arises from the epithelium of a duct.^8^, ^9^, ^10^

In recent years, emerging evidence has demonstrated that vital genes and signalling pathways are critically involved in the tumorigenesis and progression of pancreatic cancer, such as K‐ras related proteins,^11^ Notch,^12^ Hedeghog,^13^ Wnt,^14^ F‐box proteins,^15^ PI3K (phosphatidylinositol 3‐Kinase)/Akt^16^ and mTOR (mammalian target of rapamycin).^17^ Several lines of evidence has revealed that various growth factor signalling pathways are participated in pancreatic tumorigenesis and progression, including TGF (transforming growth factor),^18^ EGF (epidermal growth factor),^19^ HGF (hepatocyte growth factor),^20^ IGF (insulin‐like growth factor),^21^ PDGF (platelet‐derived growth factor)^22^ and FGF (fibroblast growth factor).^23^, ^24^ Recently, FGF has been paid attention to pancreatic cancer development and progression. In fact, these pathways could have interplays. For example, Notch signalling activation increases FGF1‐mediated invasion in oral squamous cell carcinoma.^25^ FGF activates Ras‐MAPK pathway, leading to skin tumour induced by Pten deficient.^26^ Similarly, FGFR1 promotes activation of MAPK and mTOR pathway in palbociclib resistant non‐small‐cell lung cancer.^27^ Another study identified that FGF2 exerts tumour lymphangiogenesis via activating the Akt/mTOR/p70S6K.^28^ FGF signalling activates the expression of the sonic hedgehog receptor and Ptch2.^29^ In this review article, we will describe recent advances of FGF signalling pathway in pancreatic cancer. Moreover, we will dissect the available chemical inhibitors of FGF pathway for potential treatment of pancreatic cancer. Furthermore, we will discuss whether targeting FGF pathway is a novel therapeutic strategy for pancreatic cancer clinical management.

## 2 FGF/FGFR SIGNALLING PATHWAY

FGF, a kind of peptide molecule, has been identified to bind to its specific receptors of cell membrane and to govern cell growth. FGF is named due to its promotion of fibroblast proliferation and is located in various tissues. FGF is also called heparin conjugate growth factor because of its high affinity for heparin. At present, more than 20 members of the FGF family are identified, which are encoded by various genes.^30^ The structure of FGF protein contains heparin sulphate binding domain and FGFR binding domains.^30^ FGF1 (aFGF, acidic FGF) and FGF2 (bFGF, basic FGF) were originally thought to be potent mitogens for some cell types. FGF2 has two isoforms; the extracellular LMW isoform and predominantly nuclear HMW isoforms.^31^ Five types of FGFRs (FGFR1, 2, 3, 4, 5) have been reported and isoforms of FGFR‐1, ‐2, ‐3 have FGFR1b, FGFR1c, FGFR2b, FGFR2c, FGFR3a, FGFR3b, FGFR3c, FGFR4 and FGFR5.^32^, ^33^ Each isoform could have different location: FGFR3b is restricted to epithelial cell types, while FGFR3c is located in mesenchymal cell types. FGFR can bind to two FGF2 isoforms: LMW and HMW isoforms.^31^ FGFR proteins contain the cytoplasmic tyrosine kinase domain, a single‐pass transmembrane domain and extracellular immunoglobulin‐like domain.^34^ Interestingly, FGFR5 (also named as FGFRL1) lacks tyrosine kinase domain, which is different from the other four types.^35^

Clearly, FGFs as ligands bind to FGFRs and activate tyrosine kinase domain of FGFRs, leading to activation of FGF/FGFR signalling pathway. Interestingly, FGF1 also binds to heparin sulphate proteoglycans (HSPG), suggesting that HSPG could be a co‐receptor of FGF1. In addition, FGF1 co‐localizes with both proteoglycans CD44 and CSPG4 at the cell surface, indicating that these receptors could be storage molecular to create a reservoir of FGF1.^36^ Heparin and heparin sulphate glycosaminoglycans (HSGAGs) can stabilize FGFs against degradation.^37^ The activation of FGF/FGFR pathway regulates several downstream targets such as PI3K/Akt, MAPK (mitogen‐activated protein kinase) or PLCγ.^38^ FGF signalling pathway plays a role in a myriad of cellular biological and physiological processes such as proliferation, differentiation, survival, migration, invasion, metastasis, wound repair and angiogenesis.^30^, ^39^ FGF signalling pathway has been identified in tumorigenesis and progression in a variety of human cancers including pancreatic cancer. In the following sections, we will decipher the role of FGF/FGFR signalling pathway in pancreatic carcinogenesis.

## 3 THE ROLE OF FGF/FGFR IN PANCREATIC CANCER

### 3.1 FGF in pancreatic cancer

FGF‐1 and FGF‐2 are overexpressed in pancreatic carcinoma cells, which are associated with advanced tumour stage and shorter survival.^40^ In line with this finding, one study has demonstrated that the expression of FGF‐1, FGF‐2 and their receptors were highly increased in pancreatic adenocarcinomas compared with normal pancreatic tissue.^41^ Moreover, increased FGF and FGFR were associated with upregulation of iNOS (inducible nitric oxide synthase) and protein tyrosine nitration in pancreatic cancer tissues, predicting the potential involvement of oxidant stress in FGF pathway‐mediated pancreatic cancer development.^41^ Subsequently, this group identified that FGF‐1 signalling inhibited peroxynitrite‐induced cell death in pancreatic cancer, suggesting that FGF‐1 plays a vital role in pancreatic adenocarcinoma.^42^ Another study reported that FGF‐1 and FGF‐2 treatment led to induction of phosphorylation of E‐cadherin and beta‐catenin on tyrosine residues, resulting in an increase in cell adhesion, tubular differentiation and reduction of invasion in pancreatic cancer cells.^43^, ^44^

Twenty‐eight years ago, one study has shown that FGF‐2 at picomolar concentrations promoted cell proliferation via regulation of ornithine decarboxylase in AR4‐2J rat pancreatic cancer cell line.^45^ Moroever, more evidence has emerged to validate the role of FGF‐2 in pancreatic cancer. For exmaple, high expression of FGF‐2 was observed in PDAC, and patients with high level of FGF‐2 and VEGF (vascular endothelial growth factor) had shorter survival times.^46^ Consistently, tumour cell proliferative indices were significantly higher in pancreatic cancer cells with FGF‐2‐positive, indicating that the expression of FGF‐2 is associated with cell proliferation in pancreatic cancer.^47^ Similarly, a specific neutralizing antibody against FGF‐2 led to a 50% inhibition in cell proliferation in pancreatic cancer cells.^48^ Further, the high gradient of FGF‐2 enhanced cell invasiveness in pancreatic cancer cells, whereas inhibition of FGF pathway by anti‐FGF receptor antibody retarded cell invasion, demonstrating that FGF‐2 is involved in cell invasiveness in pancreatic cancer.^49^ Additionally, Pim‐3, a proto‐oncogene with serine/threonine kinase activity, promoted tumour neovascularization and tumour growth via upregulation of FGF‐2 in pancreatic cancer.^50^ Klotho, a transmembrane protein, suppressed cell growth in vitro and in vivo through inactivation of FGF‐2 pathway in pancreatic cancer.^51^ Interestingly, secretory FGF‐2 upregulation was exhibited to have the potential to inhibit spreading of pancreatic cancer cells.^52^

FGF‐5 has been reported to be involved in various biological processes including development, tissue growth, repair and morphogenesis.^53^ FGF‐5 was initially identified to be an oncogene in human cancers.^54^ FGF‐5 mRNA was detected in pancreatic cancer cells and secreted FGF‐5 protein was observed in conditioned medium of pancreatic cancer cells. Overexpression of FGF‐5 promoted the cell growth and increased MAPK activity in pancreatic cancer.^55^ Expression of FGFR‐1 IIIc variant mediated FGF‐5‐induced mitogenic responsiveness through the MAPK pathway in pancreatic ductal cells, indicating that FGF‐5 in conjunction with FGFR‐1 IIIc could contribute to pancreatic cancer pathobiology.^56^, ^57^

FGF‐7, also called as keratinocyte growth factor (KGF), is originally observed in mesenchymal cells, demonstrated that FGF‐7 might be involved in mesenchymal stimulation of epithelial cell proliferation.^58^ FGF‐7/KGF is frequently overexpressed in pancreatic cancer.^59^ KGF/FGF‐7 activated NF‐κB (nuclear factor kappa B) and subsequently induced the expression of VEGF, MMP‐9 and urokinase‐type plasminogen activator, leading to enhancement of migration and invasion in pancreatic ductal epithelial cells. This finding identify that KGF/FGF‐7 could be a malignancy‐contributing factor from tumour stroma.^59^ FGF10, a FGF‐7 subfamily member, exerted its biological responses via activation of FGFR2b. One study reported that FGF‐10 can participate in transmitting mesenchyme signalling to the epithelium and involved in pancreas development.^60^ Stimulation of pancreatic cancer cells with FGF‐1, FGF‐2, FGF‐7 and FGF‐10 resulted in changes in the expression of key genes such as SOX‐9 (SRY‐related HMG‐box gene 9), HNF3β (hepatocyte nuclear factor 3‐beta), GATA‐4, GATA‐6 and HES1 (hairy and enhancer of split‐1).^61^ This study suggests that these growth factors might be involved in pancreatic cancer development. FGF‐10 was observed in stromal cells surrounding the cancer cells in pancreatic cancer tissues. FGF‐10 induced cell migration and invasion through interaction with FGFR2 IIIb and increased expression level of MT1‐MMP (membrane type 1‐matrix metalloproteinase) and TGF‐β1 in pancreatic cancer.^62^ Consistently, FGF10 was significantly overexpressed in pancreatic cancer patients compared with healthy controls. FGF‐10 had differentially expressed in response to gemcitabine and erlotinib, suggesting that FGF‐10 could be a predictive biomarker for chemotherapeutic treatment response in pancreatic cancer patients.^63^

FGF‐13 was found to be significantly associated with the shorter survival and occurrence of liver metastasis in pancreatic cancer.^64^ This investigation identifies FGF‐13 as a novel prognostic biomarker in pancreatic cancer. Overexpression of FGF‐19 did not affect the cell proliferation, but inhibited cell migration, invasion and attachment via stimulation of FGFR4 in pancreatic cancer cells.^65^ Several knockout mouse phenotypes have demonstrated the role of FGFs in tumorigenesis. *Ffg15* (human homolog, FGF19) deficiency impairs liver regeneration in mice.^66^ Moreover, fibrosis‐induced hepatocellular carcinoma development is retarded in *Fgf15* knockout mice.^67^ Inducible Fgf13 ablation in cardiomyocytes enhances caveolae‐induced cardioprotection during cardiac pressure overload.^68^ Loss of *Fgf21* leads to insulin resistance, pancreatic islet hyperplasia and dysfunction in mice. *Fgf23* knockout mice impair the auditory system and the metabolism of phosphate and active vitamin D in the kidney.^69^, ^70^ *Fgf19* transgenic mice developed hepatocellular carcinomas.^71^ Transgenic expression of FGF8 and FGF10 results in the development of hepatocytes and exocrine cells from pancreatic islet cells transdifferentiation.^72^ Prostate‐targeted *Fgf8b* transgenic mice have stromal activation and prostate cancer development.^73^ Fgf‐2 transgenic mice have glandular epithelial hyperplasia in the murine prostatic dorsal lobe.^74^ Without a doubt, the engineering mouse model is an ideal vehicle for studying the role of FGF in human cancers including pancreatic cancer.

### 3.2 FGF‐binding proteins in pancreatic cancer

FGF‐binding proteins (FGF‐BP) release FGFs from the extracellular matrix storage, leading to increased FGF activity. Therefore, FGF‐BP plays a critical role as an extracellular chaperone in FGF‐mediated signalling pathway and mitogenesis.^75^, ^76^, ^77^ Moreover, FGF‐BP expression is remarkably increased in a variety of human cancer tissues.^78^ FGF‐BP1 expression is highly elevated in pancreatic adenocarcinoma compared with normal pancreas, suggesting that FGF‐BP1 might a biomarker for high‐risk premalignant lesions.^79^ In consistent, FGF‐BP1 was found to be induced early during the pancreatic cancer initiation.^80^ These reports clearly indicate that FGF‐BP could become an indicator of early diagnosis for pancreatic cancer. The results from *Fgfbp3* knockout mice showed that FGF‐BP3 impacts carbohydrate and lipid metabolism.^81^ To further investigate the role of FGF‐BP in tumorigenesis, *Fgf‐bp* engineering mice are required.

### 3.3 FGFR in pancreatic cancer

Twenty‐five years ago, aberrant expression of FGFR1 was observed in pancreatic cancer.^82^ Moreover, the 2‐immunoglobulin‐like form of FGFR1 was reported to involve in aberrant autocrine and paracrine pathways in pancreatic cancer.^82^ One study showed that inhibition of FGFR‐1 decreased cell growth in vitro and retarded tumour‐forming potential in vivo in pancreatic cancer. Moreover, FGF/FGFR‐1 exerted its function via regulation of receptor tyrosine phosphorylation and MAPK activation in pancreatic cancer.^83^ Overexpression of FGFR‐1α increased cell death via activation of caspase 3 and inhibition of Bcl‐xL (B‐cell lymphoma‐extra large)/BAX in pancreatic cancer cells. Moreover, FGFR‐1α overexpression suppressed cell growth and restored cytotoxic responses to chemotherapy.^84^ However, overexpression of FGFR‐1β led to formation of tumour xenograft and exhibited resistance to chemotherapy.^84^ Liu et al found that FGFR1 IIIb suppressed the formation and growth of tumours in mice, which have a reduced Ki‐67 and a lower level of tumour necrosis in tumours. This study showed that FGFR1 IIIb blocks the transformation phenotype of pancreatic cancer cells.^85^ Another study revealed that FGFR1 IIIb overexpression promoted the expression of SPARC (secreted protein acidic and rich in cysteine), which is a protein‐modulating cell–cell and cell–matrix interactions. FGFR1 IIIc overexpression decreased SPARC level in pancreatic cancer cells.^86^ This suggests that FGFR1‐III isoforms exert their function partly via modulation of SPARC expression in pancreatic cancer.

The FGFR1 IIIb induced cell proliferation after FGF‐1, FGF‐2 and FGF‐4 stimulations via production of a glycosylated 110kd protein in pancreatic cancer cells. The FGF‐1, FGF‐2 and FGF10 induced activation of MAPK and c‐Jun N‐terminal kinase and led to cell proliferation enhancement. Moreover, the FGFR1 IIIb increased single‐cell movement and plating efficacy. Thus, the FGFR1 IIIb could govern cell proliferation, adhesion and movement in pancreas.^87^ Blockade of FGF‐2‐induced proliferation of pancreatic cancer cells by an adenoviral vector encoding a truncated FGFR‐1 (AdtrFGFR‐1) led to decreased MAPK activation, implying that AdtrFGFR‐1 could be useful as a therapeutic agent in pancreatic cancer.^88^ Similarly, a recombinant adenovirus expressing soluble FGF receptor (AdsFGFR) suppressed tumour angiogenesis and tumour growth in vitro and in vivo, indicating that FGFR plays a key role in tumour angiogenesis.^89^ Clinically, high expression of FGFR was associated with the extent of malignancy and post‐operative survival in human PDAC.^90^

FGFR2 expression was observed in pancreatic cancer cells. Patients with high level of FGFR2 exhibited a shorter survival time in pancreatic cancer.^62^ Downregulation of FGFR‐2 by its shRNA infection targeting the IIIb and IIIc isoforms inhibited cell proliferation, migration and invasion in PDAC cells. Additionally, downregulation of FGFR‐2 led to decreased phosphorylation of ERK (extracellular signal‐regulated kinases) and VEGF‐A in PDAC cells after FGF‐2 stimulation. Moreover, inhibition of FGFR‐2 resulted in smaller tumours in nude mice, suggesting that FGFR‐2 could be a potential target for pancreatic cancer.^91^ Similarly, inhibition of FGFR signalling using shRNA led to cell kill in pancreatic cancer cells. Dovitinib treatment in combination with FGFR shRNA transfection achieved significant anti‐tumour effects in pancreatic cancer, especially in FGFR2 IIIb overexpressing pancreatic cancer cells.^92^ Furthermore, FGFR2 IIIc was highly expressed in PDAC tissues, which is associated with liver metastasis in PDAC patients. In line with the role of FGFR2 in PDAC, overexpression of FGFR2 IIIc promoted cell proliferation in vitro and enhanced tumour growth and live metastases in vivo via upregulation of p‐ERK (phosphorylated extracellular signal‐regulated kinase) in PDAC.^93^ One study showed that targeting the CYP2B1/cyclophosphamide suicide system to FGFRs led to tumour suppressive response and an increased survival rate in pancreatic cancer.^94^

FGFR4 was expressed in a majority of pancreatic cancer patients, and its expression was related to longer overall survival. FGFR4 stimulation led to increased cell adhesion to laminin and fibronection, and inhibited cell migration, suggesting that FGFR4 could contribute to tumour suppressive function via enhanced cell adhesion to extracellular matrix.^65^ Consistently, dominant‐negative FGFR‐4 and inhibitors of FGFR signalling inhibited matrix adhesion induced by N‐CAM (neural cell‐adhesion molecule) in pancreatic cancer. Moreover, N‐CAM promoted β1‐integrin‐involved cell–matrix adhesion via activation of FGFR signalling pathway.^95^ Additionally, FGFR4 knockout mice bred with FGF19 transgenic mice fail to develop liver tumours.^71^ The engineering mice are necessary to explore the function of FGFR in tumorigenesis.

## 4 FGFR INHIBITORS FOR PANCREATIC CANCER TREATMENT

Several FGFR inhibitors have been discovered for potential treatment of human cancers including pancreatic cancer^96^ (Table 1). For example, SSR128129E is an orally effective allosteric FGFR inhibitor, which has no effect on other related RTKs. Chemical SSR128129E (SSR) inhibits responses mediated by FGFR1‐4. SSR was reported to inhibit the proliferation and migration of pancreatic tumour cell line in response to FGF‐7.^97^ Dovitinib, formerly known as TKI258, a tyrosine kinase inhibitor to FGFRs, PDGFRβ (platelet‐derived growth factor receptor beta) and VEGFR2, inhibited activation of signalling intermediates in pancreatic cancer cells upon FGF‐1 and FGF‐2 treatment. TKI258 repressed surviving level, enhanced activity of gemcitabine and reduced motility of pancreatic cancer cells. Moreover, TKI258 inhibited tumour growth and lymph node metastases in mouse model, suggesting that TKI258 could be an effective agent for human pancreatic cancer.^98^ Dovitinib treatment exhibited pro‐apoptotic effect in pancreatic cancer cells with heightened FGFR signalling activation via regulation of Akt/Mcl‐1 axis.^92^ Recently, a phase 1b study showed that dovitinib with gemcitabine and capecitabine achieved efficacy signals in advanced pancreatic cancer.^99^

**Table 1:** FGFR inhibitors in cancer treatment

Inhibitors	Targets	Function	Adverse events	Ref.
BGJ398	FGFR1‐3	Inhibits cell proliferation; exerts anti‐tumour activity in several tumour types including lung cancer, bladder, urothelial cancers, cholangiocarcinoma	Hyperphosphatemia, constipation, decreased appetite, diarrhoea, fatigue, alopecia, nausea in patients	109, 124, 125, 126, 127
SSR128129E	FGFR1‐4	Inhibits proliferation, angiogenesis and metastasis in pancreatic, breast and colon cancer cells	A therapeutic dose minimally elevated plasma levels of the prothrombotic PAI‐1, a minor anaemia in mice	97
Dovitinib (TKI258)	FGFR, PDGFRβ, VEGFR2	Inhibits tumour growth, motility and metastasis; enhances the therapeutic effect of gemcitabine and capecitabine	Fatigue, neutropenia, thrombocytopenia, anaemia, nausea, palmar‐plantar erythrodysesthesia syndrome in patients	98, 99
Lenvatinib	FGFR1‐4, KIT, RET, VEGFR1‐3, PDGFRα	Inhibits tumour growth, angiogenesis in pancreatic cancer, hepatocellular cancer and melanoma	Hypertension, palmar‐plantar erythrodysesthesia syndrome, decrease appetite, proteinuria, fatigue, nausea	100, 128, 129
Masitinib	c‐Kit, FGFR and PDGFR	Inhibits inflammation, combined with gemcitabine exhibited synergy on proliferation inhibition	Back pain, constipation, pulmonary embolism, vomiting, nausea, rash, thrombocytopenia, thrombosis, hypokalemia, pyrexia, neutropenia and anaemia	101, 102, 103, 104
PD173074	FGFR1, VEGFR2	Blocks the proliferation and induces apoptosis. Inhibits stem cell proliferation and self‐renewal	No body weight loss and appearance change in mice	105, 108, 130
Nintedanib	VEGFR1/2/3, FGFR1/2/3, PDGFRα/β	Inhibits cell proliferation, induces apoptosis, enhances gemcitabine, or afatinib, or docetaxel, or cisplatin inhibitory effect	Diarrhoea, asthenia, nausea, vomiting, anaemia, anorexia, hepatic enzyme elevation, hypertension, hypothyroidism, hand‐foot syndrome, cardiac disorder, haematological abnormalities. Nintedanib plus docetaxel leads to sepsis, pneumonia, respiratory failure and pulmonary embolism	110, 111, 112, 113, 131, 132, 133, 134, 135, 136, 137, 138, 139, 140
Ponatinib	FGFRs, Bcr‐Abl, Src, PDGFRa, VEGFR2	Anti‐tumour activity in leukaemia. Combines an MEK inhibitor to inhibit pancreatic cancer cell growth	Hypertension, myelosuppression, cerebrovascular, vaso‐occlusive disease, lipase and rash	115, 141, 142, 143, 144, 145

Lenvatinib, an oral inhibitor of multiple RTKs targeting FGFR1‐4, VEGFR1‐3, PDGFRα, RET and KIT. One study has shown that lenvatinib suppressed in vivo angiogenesis induced by overexpressed FGF in pancreatic cancer. Notably, lenvatinib also inhibited tumour growth in tumour xenograft models. This report indicates that lenvatinib inhibited FGF‐ and VEGF‐driven angiogenesis in pancreatic cancer.^100^ Masitinib, a tyrosine kinase inhibitor of several targets, inhibits c‐Kit, FGFR and PDGFR. Masitinib could decrease inflammation in pancreatic cancer patients with increased pain scores.^101^ Masitinib and gemcitabine combination exhibited synergy in vitro on proliferation of pancreatic cancer cells.^102^ The efficacy and safety of masitinib/gemcitabine have been evaluated and shown to extend survival and median time‐to‐progression in pancreatic cancer.^103^, ^104^ PD173074, an effective inhibitor of FGFR1, inhibited neoangiogenesis and mitogenesis, induced apoptosis, leading to inhibition of orthotopic tumour growth in pancreatic cancer mouse model.^105^ In addition, PD173074 inhibited cell proliferation and self‐renewal of pancreatic cancer stem cells via suppression of Oct4, Sox‐2, Nanog, c‐Myc, XIAP (X‐linked inhibitor of apoptosis protein), Bcl‐2 and survivin. However, it has no direct evidence to show the role of FGF/FGFR in pancreatic cancer stem cells. Two papers suggest that FGF signalling and FGF10 were involved in enhancing differentiation of pluripotent stem cells into pancreatic progenitors.^106^, ^107^ Moreover, PD173074 induced cell apoptosis via upregulation of caspase‐3 and cleaved PARP (poly‐ADP ribose polymerase) in pancreatic cancer cells. PD173074 also inhibited the activation of c‐Met, Src, ERK1/2 and NF‐κB in pancreatic cancer cells.^108^ BGJ398 is an effective, bioactive FGFR1/2/3 inhibitor with low inhibitory effect on FGFR4, which inhibited cell proliferation of pancreatic cancer.^109^

Nintedanib (BIBF 1120), a triple tyrosine kinase inhibitor that targets VEGFR1/2/3, FGFR1/2/3 and PDGFRα/β signalling, inhibited tumour growth, enhanced the activity of gemcitabine and decreased metastatic burden in orthotopic pancreatic xenografts, suggesting that nintedanib could be a potent anti‐angiogenesis agent for pancreatic cancer.^110^ Moreover, nintedanib inhibited cell proliferation, induced apoptosis via blocking PI3K/MAPK activity and enhanced gemcitabine inhibitory effects in pancreatic cancer.^111^ Furthermore, nintedanib was identified as a highly effective therapeutic for neuroendocrine carcinoma of the pancreas using transgenic mouse model.^112^ Notably, nintedanib plus afatinib exhibit anti‐tumour activity with a manageable safety in pancreatic cancer.^113^ Ponatinib (AP24534) is an effective multitargeted inhibitor that act on FGFRs, Bcr‐Abl, Src kinase, PDGFRα, VEGFR2, Akt, ERK1/2 and other kinases.^114^ Ponatinib plus an MEK inhibitor were effective in inhibition of pancreatic cancer cell growth.^115^ BGJ398 is an effective, bioactive FGFR1/2/3 inhibitor with low inhibitory effect on FGFR4, which inhibited cell proliferation of pancreatic cancer.^109^ We believe that more FGFR inhibitors will be discovered for the treatment of pancreatic cancer. It is noteworthy that using these FGFR inhibitors could cause side effects on cancer patients. For instance, TKIs could lead to adverse effects on viral organs, including the cardiovascular system and liver.^116^ Hypertension is associated with the treatment of nintedanib, lenvatinib, ponatinib, cabozantinib and trametinib.^116^ Moreover, ponatinib treatment for chronic myeloid leukaemia results in cardiovascular adverse effects, such as vascular occlusive event.^117^ Due to inhibition of VEGFR by these TKIs, these inhibitors’ application could lead to bleeding and thrombosis.^118^ Hence, it is required to reduce adverse effects of FGFR inhibitors.

## 5 CONCLUSION AND PERSPECTIVE

**Figure 1:**
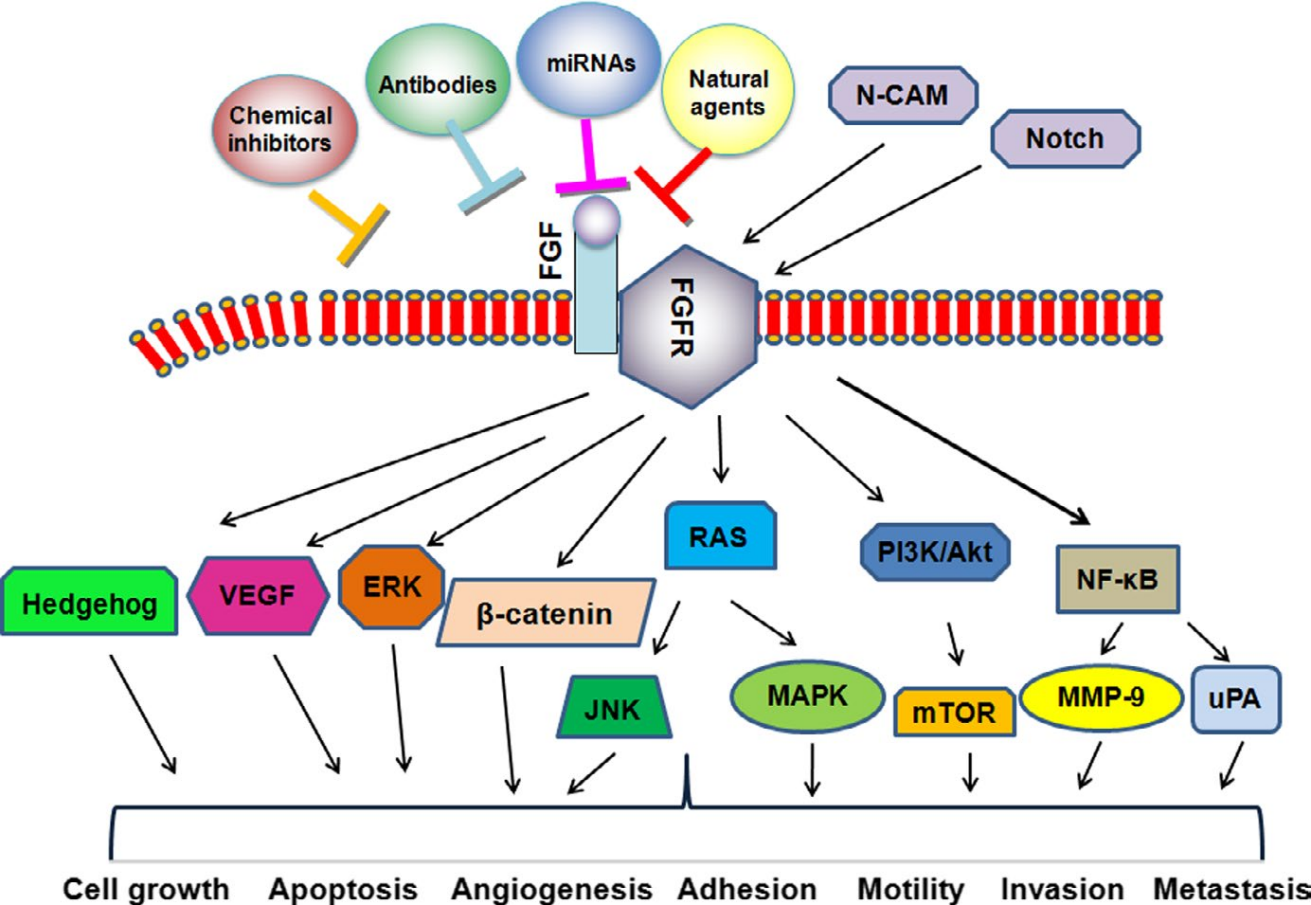
Role of the FGF/FGFR signalling pathway in the development and progression of pancreatic cancer. Fibroblast growth factor (FGF) signalling pathway regulates numerous cellular processes such as cell proliferation, apoptosis, angiogenesis, migration, invasion and metastasis. FGF/FGFR could be regulated by Notch, N‐CAM and miRNAs. FGF/FGFR exhibits its physiological functions via regulation of its downstream targets. The chemical inhibitors of FGF/FGFR, antibodies and natural agents could block FGF signalling pathway. Thus, targeting FGF/FGFR could be an effective approach for the treatment of pancreatic cancer patients

In summary, FGF plays an important role in the development and progression of human pancreatic cancer because FGF pathway is critical involved in numerous cellular processes including proliferation, apoptosis, migration, invasion, angiogenesis and metastasis (Figure 1). FGF/FGFR has been revealed to participate in its regulatory functions through regulation of its downstream targets (Table 2). Therefore, targeting FGF/FGFR pathway might be an effective strategy for treating pancreatic cancer. However, several questions should be addressed regarding role of FGF/FGFR in pancreatic cancer. Since the upstream and downstream components involved in FGF/FGFR pathway are largely unknown, it is required to identify these components that could be helpful for discovery of new inhibitor of FGFR for pancreatic cancer treatment. Because FGF/FGFR could have different roles in various organisms, it is better to find an approach for discovery of FGF/FGFR inhibitors in the specific organism with minimal effect on other organisms. Because available FGFR inhibitors target multiple molecules, which could lead to side effect function, it is better to develop the specific inhibitor for one molecule. Blocking a single FGFR with a monoclonal antibody could be helpful for cancer patients with amplification or constitutive activation of a special subtype of FGFR. Due to that most cancers with upregulation of FGFs and FGFR subtypes, targeting one FGFR by its antibody or siRNA might not acquire the treatment benefit. Recently, several microRNAs (miRNAs) have been identified to target FGF/FGFR pathway in human cancer.^119^, ^120^, ^121^ For example, miR‐214 inhibits the expression of FGFR‐1, leading to suppression of hepatocellular carcinoma metastasis.^119^ One study showed that miR‐99a targets FGFR3 in epithelial ovarian cancer cells.^120^ Another study validated the miRNA panel, including let‐7c, miR‐155 and miR‐218, could be useful for prediction of response to ponatinib in lung cancer cells.^121^ FGF2 was a direct target of miR‐186‐5p in glioblastoma multiforme.^122^ Moreover, FGF‐2 regulates cell proliferation, migration and angiogenesis via governing NDY1/KDM2B‐miR‐101‐EZH2 pathway in bladder cancer.^123^ However, studies for role of miRNAs regulating FGF/FGFR in pancreatic cancer progression are not available. How to use FGFR inhibitors in combination with chemotherapeutic drugs to maximize the treatment benefit in cancer patients? Taken together, uncovering the molecular mechanism regarding how FGF pathway is involved in pancreatic tumorigenesis would shed light onto the discovery of new effective inhibitors of FGFR.

**Table 2:** Role of FGF/FGFR in pancreatic cancer

FGF/FGFR	Targets	Function	Reference
FGF‐1	Induction of phosphorylation of E‐cadherin and β‐catenin, regulation of SOX‐9, HNF3β, HES1	Overexpression; associates with advanced tumour stage and shorter survival	40, 41, 43, 44, 61
FGF‐2	Induction of phosphorylation of E‐cadherin and β‐catenin, regulation of SOX‐9, HNF3β, HES1, ornithine decarboxylase	Overexpression; associates with advanced tumour stage and shorter survival; promotes cell growth and invasion	40, 41, 43, 44, 47, 49, 61
FGF‐5	Induction of MAPK activity	Overexpression; promotes the cell growth	55
FGF‐7	Activates NF‐κB, VEGF, MMP‐9 and uPA, regulation of SOX‐9, HNF3β, HES1	Overexpression; promotes migration and invasion	59, 61
FGF‐10	Increases MT1‐MMP and TGF‐β1, regulation of SOX‐9, HNF3β, HES1	Induces cell migration and invasion. Overexpressed; a biomarker for chemotherapeutic treatment response	61, 62, 63
FGF‐13	Not identified	Associates with the shorter survival and occurrence of liver metastasis in pancreatic cancer	64
FGF‐19	Stimulation of FGFR4	Inhibits cell migration, invasion and attachment	65
FGF‐BP1	Not identified	Overexpression; Induces early during the pancreatic cancer initiation	79, 80
FGFR‐1	Activation of MAPK, caspase 3, inhibition of Bcl‐xL/Bax and SPARC	Controls cell growth, cell death, adhesion, movement and tumour angiogenesis	83, 84, 85, 86, 87, 89
FGFR‐2	ERK, VEGF‐A	Overexpression; associates with a shorter survival rate; inhibits cell proliferation, migration and invasion	62, 91, 93
FGFR‐4	PLC‐γ, PI3K, MAPK	Associates with longer overall survival; increases cell adhesion, inhibits cell migration	65, 95

## CONFLICT OF INTEREST

The authors declare that they have no conflict of interest.

## AUTHOR CONTRIBUTIONS

All authors are involved in writing this manuscript and approved this article.
